# Identification of pre-frail/frail older adults using the Integrated Care for Older People WHO Step1 screening tool: a cross sectional study

**DOI:** 10.1016/j.ijnsa.2025.100463

**Published:** 2025-12-04

**Authors:** Caroline Berbon, Gabor Abellan Van Kan, Yves Rolland, Laurent Balardy, Catherine TAKEDA, Néda Tavassoli, Véronique Bezombes, Bruno Vellas, Sandrine Andrieu, Maria Eugenia Soto

**Affiliations:** a IHU HealthAge & WHO Collaborating Center for Frailty, Clinical & Geroscience Research, and Geriatric Training, Toulouse University Hospital, La Grave, 31 059 Toulouse, France; b Maintain Aging Research team, Centre d’Epidémiologie et de Recherche en Santé des Populations, Université de Toulouse, Inserm, Université de Toulouse, 31 400 Toulouse; c Clinical Epidemiology and Public Health Department, Toulouse University Hospital, allées Jules-Guesde, 31000 Toulouse, France

**Keywords:** Frailty, Integrated care, Prevention, Healthcare professionals, WHO

## Abstract

**Background:**

The Integrated Care for Older People program is a recommendation from the World Health Organization (WHO) to prevent functional decline. It is carried out in four steps, including Step1 screener, Step2 comprehensive assessment in case of anomalies detected in Step1, Step3, which is a prevention plan and Step4, involving long-term follow-up of intrinsic capacity and the implementation of the care plan. In order to meet this evaluation, need and given the constraints of healthcare professional, it is important to specify which older people are most at risk of decline (pre-frail/frail people according to Linda Fried's score).

**Objective:**

to assess if Integrated Care for Older People WHO Step1 can segregate pre-frail/frail older adults from robust in primary care setting.

**Method:**

This cross-sectional study concerned participants registered on the ICOPE MONITOR platform of the Toulouse University Hospital. The results from Step1, which help identify participants most at risk of pre-frailty/frailty, were selected through regression analysis followed by a Classification and Regression Trees approach.

**Results:**

Between January 1, 2020, and June 30, 2024, 3,118 participants were included in this study. Pre-frailty/frailty was associated with mobility alert alone (84.7 %), nutrition alert alone (88.9 %) and with the two associated (96.7 %). For the oldest participants (over 76 years), a psychology alert was also strongly associated with pre-frailty/frailty (63.9 %).

**Discussion:**

This study allows healthcare professional to prioritize participants with impaired mobility and/or nutrition at Step1 for the next steps in Integrated Care for Older People program. This approach addresses the needs of participants by providing the appropriate response to the right clinical profile and facilitates the interpretation of Integrated Care for Older People Step1 in the community.

**What is already known**

• Identifying pre-frail/frail older people in clinical practice is essential to prevent functional decline and dependency
• Validated tools for identifying pre-frail/frail older people are rarely used in clinical practice because they are too complex or time-consuming
• Step1 of the Integrated Care for Older PEople program helps identify the risk of intrinsic capacity decline, but its ability to detect pre-frail/frail older people — those most at risk of tipping into dependency — still needs to be evaluated

Alt-text: Unlabelled box

**What this paper adds**

• Nurses are key players in the implementation of the Integrated Care for Older PEople (ICOPE) program, especially among the frailest older people
• Step1 of the ICOPE program may be useful in routine clinical practice for identifying pre-frail/frail older people
• Older people at highest risk of functional decline are those with impaired mobility and/or nutrition at Step1, and should be prioritized for further care within the ICOPE pathway

Alt-text: Unlabelled box

## Introduction

The Integrated Care for Older People (ICOPE) program (WHO, 2024), recommended by the World Health Organization (WHO), is intended to be offered to as many adults aged 60 and over as possible, with the goal of preventing declines in intrinsic capacity and thereby maintaining functional autonomy (WHO, 2024). Since WHO recommendation for healthy Aging, Integrated Care for Older People program is proposed to all older adults—whether robust, pre-frail, or frail—to monitor changes in their intrinsic capacity (WHO, 2024) and provide tailored interventions in case of a confirmed decline of one or several domains of their intrinsic capacity (nutrition, sensory, mood, cognition, mobility). Among these older adults some are pre-frail and frail individuals that may be prioritized for intervention. Pre-frailty and frailty are clinical syndrome with a clinical status that combined at least one abnormality among slowness, weight loss, sedentarism, fatigue, weakness. Pre-frail and frail individuals remain independent but show a reduced intrinsic capacity (Gonzalez-Bautista et al., 2020, Wang et al., 2024), with impairments in locomotion and nutrition particularly associated with pre-frailty and frailty (Liu et al., 2021). As frail individuals have a reduced ability to respond to stressors (Rolland et al., 2011), they are at high risk of dependency (Wang et al., 2024). For this reason, frail individuals should be the primary target for intervention. With the global population aging (WHO, 2024), identifying pre-frail and frail older adults has become a major challenge and Integrated Care for Older People program should facilitate the targeting of this population.

Integrated Care for Older People program has been piloted in Occitanie, France since 2020 with the support of the Regional Health Agencies and the Ministry of Health (Tavassoli et al., 2021). Intrinsic capacity is defined by six domains—cognition, psychology, mobility, nutrition, vision, and hearing—all of which interact with an individual’s environment and various health conditions (WHO, 2024). Our experience with Integrated Care for Older People program implementation shows that the involvement of healthcare professionals—including nurses, family doctors, physiotherapists, and pharmacists—is essential (Tavassoli et al., 2021) (Tavassoli et al., 2022) for the successful execution of the program. The WHOIntegrated Care for Older People Step1, a simple, low-cost, and time-efficient screening tool, has already been shown to be reliable for identifying impairments in intrinsic capacity (Giudici et al., 2024 Jul 1). Step1 can be conducted by healthcare professionals, non-healthcare professionals, through self-assessment, or with the help of a proxy. In contrast, the subsequent steps of the program require the expertise of trained healthcare professionals (Berbon et al., 2025) to confirm a decline in intrinsic capacity (Step1), perform a comprehensive assessment if a decline is detected (Step2), develop an individualized care and prevention plan (Step3), and monitor the implementation of the care plan (Step4) (Tavassoli et al., 2022). Once Step1 is completed, a healthcare professional reviews the results and, if the decline is deemed clinically relevant, recommends a Step2 assessment. The Step2 assessment and subsequent steps require the training and involvement of numerous healthcare professionals working in the community or in private and public institutions. The successful implementation of Integrated Care for Older People program also depends on training tools, structured care pathways, and telemonitoring platforms (Tavassoli et al., 2022). In this process, primary healthcare professionals ensure that the Integrated Care for Older People program is delivered as close as possible to participants’ living environments, particularly in isolated areas with limited healthcare resources. These healthcare professionals are primarily nurses. They initiate the Integrated Care for Older People program for those over 60 and facilitate both their monitoring and their adherence to the program when an alert appears. In subjects who immediately have an alert, it is important to identify those most at risk of dependency in order to prioritize their efforts (Berbon et al., 2024). In addition to their regular care duties and within the context of a limited healthcare workforce, they must prioritize Step2 assessments for participants at the highest risk of functional decline and progression to dependency. Integrated Care for Older People program is the global program supported by the WHO to contribute to healthy aging. Integrated Care for Older People Step1 has demonstrated high sensitivity (Berbon et al., 2025), and feasibility making it effective for identifying individuals at risk of functional decline by screening across all domains of intrinsic capacity. It consists of validated questions and tests and is feasible in routine clinical practice (WHO, 2024). Therefore, a robust individual may show initial abnormalities in Step1. Providing preventive recommendations to them is essential, but a major challenge of Integrated Care for Older People program is to target the population most in need of prevention when Step1 is abnormal. Frailty is widely recognized as a clinical condition associated with a high risk of disability (Rolland et al., 2011), and the Linda Fried criteria have been extensively used in research (Fried et al., 1991) to prevent disability (Fried et al., 2001). The Fried’s scale is complicated to implement in clinical practice. I is rarely used in routine clinical practice due to its complexity. Other frailty detection tools exist (Buta et al., 2016), but they are either poorly validated, complex, or time-consuming (Rockwood et al., 2005, Searle et al., 2008). The ability of Integrated Care for Older People step 1 to detect pre-frailty and frailty should make it possible to prioritize the implementation of strategies to prevent functional decline. Therefore, Step1 could represent a valuable alternative, enabling primary care healthcare professionals to provide simple, practical clinical recommendations.

Our hypothesis is that Step1 of the WHO Integrated Care for Older People program can identify pre-frailty and frailty (defined according to the Fried criteria (Fried et al., 1991)). Identifying pre-frail and frail individuals among those with an abnormal WHO Integrated Care for Older People Step1 could help prioritize the implementation of Step 2 and 3 for participants at the highest risk of functional decline. The main objective of our study is to assess whether WHO Integrated Care for Older People Step1, age and gender can distinguish pre-frail and frail older adults from robust individuals in a primary care setting.

## Methods

### Design

This cross-sectional study concerned participants registered on the ICOPE MONITOR platform of the Toulouse University Hospital between January 1, 2020, and June 30, 2024.

### Ethical considerations

The ICOPE MONITOR database complies with data confidentiality and security requirements in accordance with the General Data Protection Regulation (GDPR) and has been validated by the National Commission on Informatics and Liberties (CNIL) in 2017 (registration number 247169284s, reference MMS/OSS/NDT171027). After evaluation and validation by the data protection officer and according to the GDPR, this study completing all the criteria, it is register in the register of data study of the Toulouse Hospital (number’s register: RnIPH 2023-122) and cover by the MR-004.

### Participants

Participants had to be enrolled through one of the French centers using the ICOPE MONITOR system. Recruitment took place at participants’ place of residence during program information sessions, during visits to their general practitioner or another healthcare professional, through self-assessment following an information campaign, or, in some cases, during hospital consultations. All participants included in the study first underwent a Step1 screening assessment, followed by a Step2 in-depth evaluation. As part of the Step2 assessment, Linda Fried’s phenotypic score (Fried et al., 1991) was administered to all participants. No sample size estimation was conducted, because this work is based on real-life data during the implementation of Integrated Care for Older People program in France. All individuals meeting the inclusion and exclusion criteria were included. The ICOPE MONITOR database now comprises a sufficiently large number of participants to enable studies with substantial sample sizes (Tavassoli et al., 2022) (Berbon et al., 2025).

• Inclusion criteria

The inclusion criteria were: age 60 years or older (as specified by the WHO program). The Integrated Care for Older People program targets individuals who are independent in activities of daily living. Since this study focuses on the prevention of dependency, participants had to be independent at the time of inclusion. Independence was defined as the ability to perform activities of daily living, according to the Katz scale (Katz et al., 1963). A score of at least 5 out of 6 on the Katz scale was required to classify an individual as independent. For this study, only participants with complete data for Step1 and for the Fried phenotypic score (Fried et al., 1991) assessed at Step2 were included.

• Exclusion criteria

The exclusion criteria were participants younger than 60 years, those dependent in activities of daily living (Katz scale score < 5/6), and/or those with missing data for the Fried phenotypic score (Fried et al., 1991) assessed at Step2.

### Procedure

Participants were registered in the ICOPE MONITOR database as part of the deployment of the Integrated Care for Older People program in France (Tavassoli et al., 2021) (Tavassoli et al., 2022), either by trained healthcare professionals, by individuals involved in supporting older adults (e.g., teleassistance providers, social services, retirement funds), or by the participants themselves or their proxies following promotional activities for the Integrated Care for Older People program. Participants first underwent a Step1 screening to detect potential declines in intrinsic capacity. The screening results were analysed by a healthcare professional, who then proposed a Step2 assessment if warranted. At this stage, healthcare professionals first verified the accuracy of the Step1 alert (i.e., whether the recorded data are correct) by questioning the participant about the test administration. If the alert was confirmed, the nurses assessed the clinical impact of the impairment on the participant’s daily life using decision-making algorithms developed by the Toulouse University Hospital team. They also determined whether the issue was new, previously unknown, or not being monitored. Finally, they ensured that the participant agrees to continue with the program. Step2 assessments were conducted by trained healthcare professionals (nurses, psychologist, dietician…, who recorded the results in the ICOPE MONITOR platform. Step2 relies on validated scales and tests selected by experts in the field during the design of the program lead by WHO (WHO 2024). At each step, intrinsic capacity is evaluated across six domains: cognition, psychology, nutrition, mobility, vision, and hearing (Cesari et al., 2018).

• Step1 (supplementary data 1)

Professionals administered Step1 with the participant using the ICOPE MONITOR app or the website (www.icopemonitor.fr) which allows responses to each question or test across all domains of intrinsic capacity to be recorded. Step1 is also available in a self-assessment format within the same digital tools. In this case, participants registered in ICOPE MONITOR and completed the test independently or with the assistance of a relative. The application or website provides explanations for each question or test included in Step1. For **cognition**, memory complaints were recorded, temporal orientation was tested, and a three-word recall task was administered, as in the Mini-Mental State Examination (Kalafat et al., 2003). For **psychology**, the first two questions of the Patient Health Questionnaire-9 (Kroenke et al., 2001) were used: over the past two weeks, have you been bothered by: 1) feeling down, depressed, or hopeless? and 2) little interest or pleasure in doing things? For **mobility**, the five-chair stand test was performed, as in the Short Physical Performance Battery by Guralnik et al. (Guralnik et al., 1994). For **nutrition**, weight, weight loss over the past three months, and reported loss of appetite were recorded. For **vision**, participants were asked whether they experience any difficulties seeing with their glasses, whether they had any eye diseases, or whether they were undergoing treatment for diabetes or hypertension. For **hearing**, in hetero-assessment, a whisper test was performed. The professional whispered four two- or three-syllable words behind the participant, at a distance of approximately 60 cm (≈2 ft), first in one ear and then in the other. The participant had to correctly repeat at least three words for the test to be considered valid. If the participant used hearing aids, the test was performed with them. In self-assessment, only the participant’s or relatives’ reports of hearing loss were collected.

• Step2

Step2 was conducted by a trained healthcare professional, most often a nurse, who performed an in-depth assessment of the intrinsic capacity domains in which abnormalities were identified during Step1. Step2 usually took place in the participant’s home, but may also be carried out in a medical office or hospital. The tests used are validated for older adults aged 60 years and older. As part of Step2, the Fried score (Fried et al., 1991) was assessed. It included five criteria: 1) Unintentional weight loss: the healthcare professional asked whether the participant had unintentionally lost at least 4.5 kg over the past 12 months. One point was scored if the answer was “yes” (0 for no). 2) Self-reported exhaustion: the participant was asked how often during the past week they felt “everything I did was an effort” and/or “I could not get going.” The criterion was considered present (one point) if the participant reported at least one of these states “often” or “most of the time.” 3) Handgrip strength: measured using a hand dynamometer. The participant may have performed three trials with each hand, and the highest value was recorded. The criterion was met (one point) if grip strength (in kg) was below a threshold determined by sex and body mass index (BMI). 4) Walking speed: gait speed was measured over a distance of 4 meters. One point was scored if the participant took more than 5 seconds to walk the 4 meters (speed < 0.8 m/s). 5) Physical activity level: the participant was asked about the duration and intensity of their weekly physical activity. One point was scored if the participant reported no physical activity or described themselves as mostly sedentary. The total score ranged from 0 to 5. A score of 0 indicated robustness; 1–2 points indicated pre-frailty; and 3 or more points indicated frailty.

### Measures

• Collected data

The collected data included age and gender.

For Step1:

The data included the screening test responses for each domain of the intrinsic capacity: **Cognition**: memory complaints (yes/no), three-word recall score (Gonzalez-Bautista et al., 2020), and temporal orientation score (0–4; one point each for year, month, day, and day of the week). **Psychology**: feelings of depression during the past two weeks (yes/no) and loss of interest in activities (yes/no). **Mobility**: time required (in seconds) to complete the five-chair stand test. **Nutrition**: evidence of weight loss (yes/no) and evidence of loss of appetite (yes/no). **Vision**: presence of vision problems, ophthalmological diseases, or use of antihypertensive or antidiabetic treatments, as well as recent complaints of vision loss (yes/no to at least one of these items). **Hearing**: successful performance on the whisper test for each ear (yes/no), or presence of a self-reported hearing complaint (yes/no) in self-assessment. These variables determine the presence of an alert in each domain of intrinsic capacity. For each domain, if abnormalities are recorded in the ICOPE MONITOR app or on www.icopemonitor.fr the data are stored in the database and generate an alert (e.g., presence of a cognitive alert: yes/no).

For Step2:

The Step2 data included the number of comorbidities, the number of medications, and whether participants lived alone. For each domain of intrinsic capacity, the score of a scientifically validated scale or test was collected: **Cognition**: The Mini-Mental State Examination (Kalafat et al., 2003), ranging from 0 to 30, with higher scores indicating better cognitive status. This test is internationally validated and available in French. Its reliability is approximately 0.90 for both test–retest and inter-rater assessments, and its sensitivity ranges from 70 % to 90 % depending on the threshold used (Mitchell, 2009). **Psychology**: The Patient Health Questionnaire-9 (Kroenke et al., 2001), ranging from 0 to 27, with higher scores indicating more severe depressive symptoms. This questionnaire covers the main symptoms of depression, is strongly correlated with other depression scales, has a reliability of 0.84–0.89 depending on the criterion, and a sensitivity of 88 % (Levis et al., 2019). **Mobility**: The Short Physical Performance Battery (Guralnik et al., 1994), ranging from 0 to 12. A score < 6 indicates low performance, 7–9 indicates medium performance, and 10–12 indicates good performance. This test has an intra-rater reliability of 0.88 and inter-rater reliability of 0.86. Its sensitivity varies depending on the reference physical performance measure but is generally good (Levis et al., 2019) (Eusepi et al., July 18, 2025). Fried’s Frailty Phenotype (Fried et al., 1991), consisting of five criteria. Participants are classified as robust (0 criteria), pre-frail (1–2 criteria), or frail (≥ 3 criteria). This phenotype has demonstrated good validity and reliability across different settings, strong predictive validity for disability, hospitalization, and mortality, and good inter- and intra-rater reliability (Fried et al., 2001) (Bandeen-Roche et al., 2006). The Lawton Instrumental Activities of Daily Living Scale (Graf, 2008), ranging from 0 to 8, with higher scores indicating greater independence. Its reliability and validity vary across studies but are generally rated as good to excellent (Graf, 2008). **Nutrition**: Body Mass Index (BMI; weight quoted by height²). The Mini-Nutritional Assessment (Lilamand et al., 2015), ranging from 0 to 30. Scores < 17 indicate malnutrition, 17–23.5 indicate risk of malnutrition, and ≥ 24 indicate normal nutritional status. This assessment demonstrates good to excellent reliability (intraclass correlation coefficient 0.70–0.95) and strong validity, correlating with anthropometric measures, clinical assessments, and predicting morbidity and mortality in older adults (Vellas et al., 1999) (Vellas et al., 2006). **Vision**: the number of participants with abnormal distance and near vision according to the World Health Organization’s simple visual acuity scale (WHO 2024). **Hearing**: The Hearing Handicap Inventory for Older People (Duchêne et al., 2022), ranging from 0 to 40, with higher scores indicating greater hearing difficulties. It demonstrates good reliability (Cronbach’s alpha 0.87–0.91; intraclass correlation coefficient 0.84–0.85) and good validity, correlating with audiometric thresholds, quality of life, and depression measures (Ting and Huang, 2023).

• Study data

The population was dichotomized based on frailty status according to the Fried score (Fried et al., 1991). Participants with at least one of the five Fried criteria (pre-frail and frail) were compared to those with no positive criteria (robust). The groups—pre-frail, frail and robust—are described by mean age and the number of women. For the ICOPE WHO Step1, variables include the total number of alerts and the presence of an alert in each of the six domains. Specifically: Cognition: presence of a memory problem, orientation score, and three-word recall score. Mobility: time to complete the five-chair stand test (in seconds). Nutrition: participant’s weight (in kilograms). An alert is defined as an abnormality in Step1 in each domain: **Cognition**: an error in orientation or in three-word recall. **Psychology**: answering “yes” to feeling depressed and/or loss of interest in activities. **Mobility**: chair stand test time > 14 seconds for participants under 80 years, or > 16 seconds for those 80 years or older. **Nutrition**: answering “yes” to weight loss and/or loss of appetite. **Vision**: answering “yes” to visual impairments, ocular pathologies, or antihypertensive/antidiabetic treatment, associated with a reported worsening over the last six months. **Hearing**: abnormal whisper test in at least one ear or self-reported hearing complaint by the participant and/or their relatives. For Step2 assessment, outcomes on the validated scales are described, providing a more precise characterization of participants and their intrinsic capacity across domains. Results from the Short Physical Performance Battery and Mini-Nutritional Assessment are also categorized: high, medium, or low for the Short Physical Performance Battery; and normal, at risk, or malnourished for the Mini-Nutritional Assessment.

### Statistical analyses

Categorial variables are described by their counts (N) and frequencies ( %). The distribution of continuous variables is characterized by their mean (X) and standard deviation (SD) or by the median and interquartile range, depending on the variable's distribution. The included population and the population not included due to missing data were described in order to assess their differences. The comparison of characteristics between the frail population, pre-frail population and the robust population is conducted using the Chi-square test or Fisher's exact test for frequency comparisons, depending on the sample sizes. Continuous variables are compared using analysis of variance (ANOVA) or the Kruskal-Wallis test, based on the distribution of the variables. Significantly impaired domains associated with pre-frailty/frailty status, age, and gender are analysed using multivariate logistic regression modelling with a stepwise descending approach. The final model was analysed using Classification and Regression Trees (Lewis, 2000) to identify the most clinically relevant model. Using the selected logistic modelling, this machine learning technique analyses the study population by creating homogeneous groups of individuals with shared characteristics. Factors associated with pre-frailty/frailty are presented in order of importance (with the strongest associations at the top) in the form of a decision tree. For binary variables, Classification and Regression Trees identifies the determining category, while for continuous variables, it calculates the threshold value that defines a homogeneous group associated with pre-frailty/frailty. The number of terminal nodes is chosen to maintain homogeneous groups of a size relevant for clinical practice. Finally, the sensitivity and specificity of different combinations of elements identified by the Classification and Regression Trees analysis are studied to provide relevant prioritization for healthcare professionals in the field. The analyses for this study are conducted using the Stata software package (StataCorp LP, College Station, TX, USA), version 18.

## Results

**Fig. 1:**
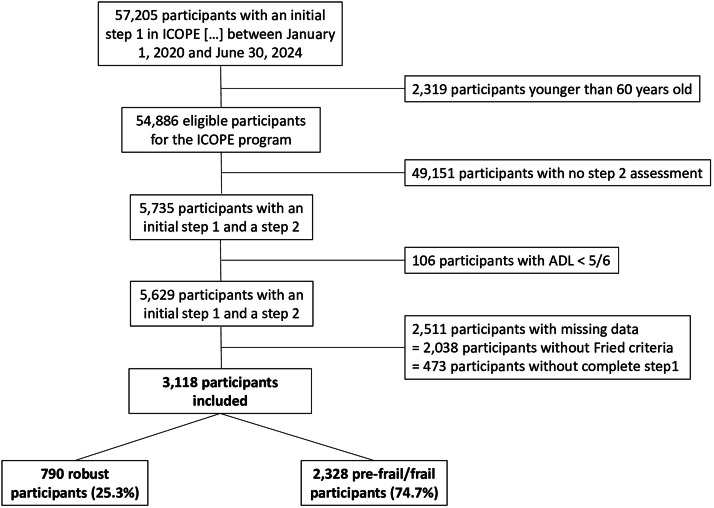
Flowchart of the study.

Between January 1, 2020, and June 30, 2024, 3,118 participants were included in this study based on the selected criteria. The flow-chart (Fig. 1) illustrates how the participants were selected. Regarding participants not included due to missing data in Step1 and Fried criteria, the analyses show a slight overrepresentation of frail individuals among the non-included participants.

Bivariate analyses: pre-frail population versus frail population versus robust population

The results of bivariate analyses are presented in Table 1. The pre-frail population and the frail population (N=2,328) appear older than the robust population (N=790) (p<0.01). Women are significantly more represented in the pre-frail and the frail populations (p<0.01). In Step1, all domains of intrinsic capacity were more alert in the pre-frail and the frail populations than in the robust population. The time taken to complete the five-chair stand test is longer for pre-frail and frail individuals (12.9±7.3 and 16.1±12.1 seconds versus 10.5±5.0 seconds; p<0.01). Pre-frail and frail individuals are more likely to live alone compared to the robust population (p<0.01). In Step2 assessment, all results for the pre-frail and the frail populations are significantly more impaired than in the robust population (except for BMI, p=0.27, and vision impairment, p=0.1). In the pre-frail population as well as the frail population, more participants had impaired physical performance on the Short physical performance battery compared to the robust population (38.8 % and 76.7 versus 12.7 %, p<0.01). Similarly, individuals at risk of malnutrition or already malnourished accounted for 24.9 % of participants in the pre-frail population and 54.8 % in the frail population compared to 7.3 % in the robust group (p<0.01). The Odds Ratio for the association of impairments in the domains of intrinsic capacity with pre-frailty/frailty are significant (p<0.01) for cognition (Odds Ratio=1.50), psychology (Odds Ratio =2.32), mobility (Odds Ratio =3.30), Nutrition (Odds Ratio =3.39) and vision (Odds Ratio =1.39). Only the impairment in the hearing domain does not have a significant association in Step1 of ICOPE with being pre-frail/frail (Odds Ratio =1.14; p=0.31).

**Table 1:** Comparison of pre-frail, frail and Robust populations, N=3,118.

				Robust (n=790)	Pre-frail (n=1,578)	Frail (n=750)	P
Age in years*				74.7 ± 7.6	78.3 ± 7.6	80.7 ± 7.6	**<0.01**
Female				501 (63·4)	1,061 (67.2)	549 (73.2)	**<0.01**
Step1	Number of alerts per subject**			2 (1 – 3)	3 (2 – 4)	3 (2 – 4)	**<0.01**
	Cognitive alert, yes			430 (54.4)	1,010 (64.0)	557 (74.3)	**<0.01**
		Cognitive impairment, yes		329 (41.6)	754 (47.8)	427 (56.9)	**<0.01**
		Score orientation /4**		3 (3 – 4)	3 (3 – 4)	3 (3 – 3)	**<0.01**
		Score recall /3**		3 (2 – 3)	3 (2 – 3)	2 (1 – 3)	**<0.01**
	Psychological alert, yes			252 (31.9)	750 (47.5)	447 (59.6)	**<0.01**
	Mobility alert, yes			131 (16.6)	539 (34.2)	444 (59.2)	**<0.01**
		Chair rise in seconds*		10.6 ± 5.0	12.9 ± 7.3	16.1 ± 12.1	**<0.01**
	Nutritional alert, yes			78 (9.9)	357 (22.6)	313 (41.7)	**<0.01**
	Vision alert, yes			319 (40.4)	740 (46.9)	371 (49.5)	**<0.01**
	Hearing alert, yes			345 (43.7)	774 (49.1)	416 (55.5)	**0.01**
Step2	Number of comorbidities**			2 (1-3)	2 (1 – 4)	3 (2 – 4)	**<0.01**
	Number of medications*			3.1 ± 2.7	4.3 ± 3.0	5.4 ± 3.2	**<0.01**
	Living alone			301 (38.1)	724 (46.2)	374 (50.4)	**<0.01**
	Cognition	MMSE* (N=2,296)		26·4 ± 3·8	25.4 ± 3.9	24.2 ± 4.1	**<0.01**
	Psychology	PHQ-9* (N=1,819)		4.0 ± 4.2	5.8 ± 5.0	8.9 ± 6.0	**<0.01**
	Mobility	SPPB** (N=2,924)		12 (11-12)	10 (8 – 12)	7 (6 – 9)	**<0.01**
		SPPB performances (N=2,924)	Good	622 (87.3)	911 (61.2)	169 (23.3)	**<0.01**
			Median	76 (10.7)	422 (28.4)	283 (39.1)	
			Bad	14 (2.0)	155 (10.4)	272 (37.6)	
		IADL**		8 (8-8)	8 (7 – 8)	7 (5 – 8)	**<0.01**
	Nutrition	BMI* in kg/m²		25.9 ± 4.6	26.3 ± 5.0	26.2 ± 5.4	0.27
		MNA* (N=2,355)		27·1 ± 2·3	25.2 ± 3.4	22.7 ± 4.1	**<0.01**
		Nutritional status MNA (N=2,355)	Normal	549 (92·7)	874 (75.1)	271 (45.2)	**<0.01**
			at risk	39 (6.6)	260 (22.3)	280 (46.8)	
			Malnutrition	4 (0.7)	30 (2.6)	48 (8.0)	
	Vision	Distant vision, abnormal N=1,568		52 (12·5)	134 (16.9)	62 (17.1)	0.096
		Near vision, abnormal N=1,581		72 (17.4)	151 (18.9)	85 (23.1)	0.114
	Hearing	HHIE-S**, N= 1,468		2 (0-10)	4 (0 – 10)	6 (0 – 12)	**0.0002**

Data is presented by * mean ± SD or n ( %) or **median (p25-p75) otherwise n ( %)

MMSE: Mini-Mental Status Examination, score from 0 to 30, PHQ-9: Patient Health Questionnaire-9, score from 0 to 27, SPPB: Short Physical Performance Battery, score from 0 to 12 (good performance score from 10 to 12, median performance score from 7 to 9, bad performance score from 0 to 6), Fried phenotype, score from 0 to 5 (0 = not frail; 1 – 2 pre-frail; 3 or more frail), IADL: Lawton Instrumental Activity of Daily Living, score from 0 to 8, MNA: Mini Nutritional Assessment, score from 0 to 30 (normal status from 24 to 30; at risk from 17 to 23.5; and malnutrition for MNA under 17), BMI: Body Mass Index, HHIE-S: Hearing Handicap Inventory for older people, score from 0 to 40.

### Multivariate logistic regression model

Table 2 shows the final result of the multivariate logistic regression model obtained through a stepwise approach. Adjusted on age and gender, the independent variables associated with pre-frailty/frailty include: cognitive alert, psychological alert, mobility alert, nutritional alert, and visual alert. The alteration of intrinsic capacity is significantly associated with pre-frailty/frailty starting from two impaired domains at Step1.

**Table 2:** Association between Step1 variables and pre-frailty/frailty, N=3,118.

Multivariate model				
		Odds Ratio	P	CI 95%
Cognitive alert*, yes		1.26	0.013	[1.05 ; 1.51]
Psychological alert*, yes		1.80	<0.01	[1.50 ; 2.17]
Mobility alert*, yes		2.83	<0.01	[2.28 ; 3.50]
Nutritional alert*, yes		2.74	<0.01	[2.11 ; 3.57]
Visual alert*, yes		1.28	0.007	[1.07 ; 1.52]
Hearing alert*, yes				
Bivariate association with each number of impaired domains				
Number of impaired domains**	1	1.20	0.289	[0.85 ; 1.69]
	2	2.00	<0.01	[1.44 ; 2.80]
	3	2.80	<0.01	[1.99 ; 3.93]
	4	5.66	<0.01	[3.83 ; 8.36]
	5	9.78	<0.01	[5.60 ; 17.06]
	6	55.22	<0.01	[7.47 ; 407.9]

Age and gender adjusted

the reference category is “no” * or zero**

CI: confidence interval

### Classification and regression trees

**Fig. 2:**
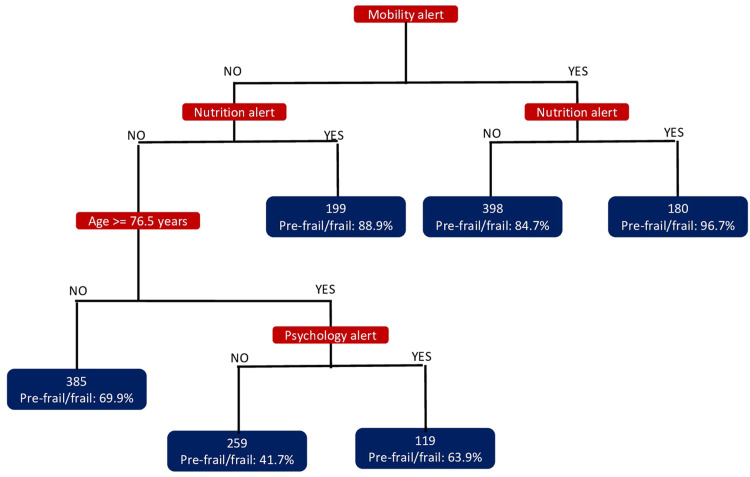
Classification and regression trees.

Based on the final multivariate model, Fig. 2 presents the retained Classification and Regression Trees model (N=1,540). In Step1, mobility alert alone is associated with pre-frailty/frailty in 84.7 % of cases; this increases to 96.7 % when mobility alert is combined with nutrition alert. Nutrition alert alone is associated with pre-frailty/frailty in 88.9 % of cases. In the absence of both mobility and nutrition alerts, the Classification and Regression Trees analysis identifies an age of 76.5 years or older combined with a psychology alert as associated with pre-frailty/frailty in 63.9 % of cases. For individuals younger than 76.5 years, the analyses do not define homogeneous groups of sufficient size with elements from Step1 of ICOPE associated with pre-frailty/frailty. Fig. 2 illustrates the predictive values of being 76.5 years or older, along with impairments in mobility, nutrition, and psychology. Table 3 shows that the sensitivity ranges from 82.5 % for being 76.5 years or older to 96.9 % for impairment in nutrition. When one domain of intrinsic capacity is impaired at Step1, sensitivity is 93.7 %, and it increases with the number of impaired domains.

**Table 3:** Predictive values of the clinical domains of Step1 associated with frailty (analyses gender adjusted).

Result at Step1	N ( %)	Sensibility	Specificity	PPV	NPV
Nutrition alert, yes	748 (24.0)	96.9	9.1	75.9	49.7
Mobility alert, yes	1,114 (35.7)	96.3	12.4	76.4	53.0
Psychology alert, yes	1,449 (46.5)	96.4	14.1	76.8	56.9
Age≥76.5, yes	1,780 (57.1)	82.5	35.8	63.1	60.6
One impaired domain	518 (16.6)	93.7	19.3	63.0	67.7
Two impaired domains	776 (24.9)	95.0	10.4	73.0	44.8
Three impaired domains	801 (25.7)	97.2	8.6	80.8	43.2
Four impaired domains	538 (17.3)	99.5	5.7	89.3	57.1
Five impaired domains	248 (8.0)	99.6	0.0	93.5	0.0
Six impaired domains	68 (2.2)	100.0	0.0	100.0	0.0

PPV: positive predictive value, NPV: negative predictive value

## Discussion

The results of this study allow the identification of pre-frail and frail older adults based on the WHO Integrated Care for Older People Step1 assessment. Our findings demonstrate that Step1 screening is an effective tool for detecting individuals who are significantly more impaired in Step2. In practice, participants presenting a mobility alert and/or a nutrition alert during Step1 are at the highest risk of pre-frailty/frailty, and consequently, at the greatest risk of disability. To a lesser extent, among the oldest participants (over 76 years), a psychology alert was also strongly associated with pre-frailty/frailty. These results could help healthcare professionals prioritize participants with abnormal Step1 results, identifying those for whom Step2 should be conducted promptly and Step3 interventions initiated as soon as possible. However, while these findings provide guidance for clinical practice, this study does not establish the overall diagnostic value of Step1 for identifying pre-frail/frail individuals, due to its very high sensitivity and low specificity.

• Link between mobility impairment in WHO Integrated Care for Older People Step1 and pre-frailty/frailty

Mobility impairment identified during WHO Integrated Care for Older People Step1 appears in our study to be strongly associated with pre-frailty and frailty. The association between mobility impairment and frailty is well-documented and has been reported in numerous studies (Avila-Funes et al., 2008). During Step1 of the Integrated Care for Older People program, mobility decline is assessed using the chair stand test, which is a reliable indicator of strength, power, and balance (Dodds et al., 2021). Impairments in these areas are closely linked to sarcopenia (Marzetti et al., 2017), a key component of frailty in older adults (Cruz-Jentoft et al., 2019) (Nascimento et al., 2019). Furthermore, several studies have shown that poor performance on the chair stand test predicts adverse health events (Musich et al., 2018) and is associated with an increased risk of mortality (Cooper et al., 2010). Prioritizing this at-risk population allows for a more in-depth evaluation of the mobility domain (Beaudart et al., 2019), considering factors such as the living environment (e.g., home modifications, accessibility, proximity of resources) and participation in a structured physical activity program.

• Link between nutrition impairment in Integrated Care for Older People Step1 and pre-frailty/frailty

This study demonstrates a strong association between nutritional impairment identified during Step1 of the Integrated Care for Older People program and pre-frailty/frailty. This association is well-established (Wysokiński et al., 2015) (Lorenzo-López et al., 2017). Integrated Care for Older People Step1 identifies nutritional decline based on two questions regarding unintentional weight loss and loss of appetite. Both factors are associated with an increased risk of malnutrition and adverse health events (Soenen and Chapman, 2013). In older adults, weight loss involves a greater proportion of muscle mass loss compared to younger adults, which can ultimately lead to sarcopenia (Soenen and Chapman, 2013). Weight loss and reduced food intake are closely linked to the development of frailty and sarcopenia (Martone et al., 2013). Addressing nutritional impairment through detailed assessment enables effective interventions, such as increasing daily protein-energy intake through dietary enrichment (Nieuwenhuizen et al., 2010)

• Association between mobility impairment and nutrition impairment

In our study, the co-occurrence of mobility impairment and nutritional impairment was associated with frailty in 96.7 % of cases. The study by Gaussens et al. has previously demonstrated the connection between nutritional and mobility impairments during Integrated Care for Older People Step 1 (Gaussens et al., 2023). This association is expected, as malnutrition leads to decreased strength, reduced muscle mass (Kaiser et al., 2024), and impaired physical performance (Cruz-Jentoft et al., 2019). Sarcopenia and the risk of malnutrition—or malnutrition itself—are strongly associated, independently of other health conditions (El et al., 2016; Calcaterra et al., 2024). The effectiveness of addressing both malnutrition and impaired physical performance has been demonstrated in the context of multimodal interventions (Nascimento et al., 2019) (Vellas et al., 2014) that combine physical activity, cognitive training, and nutritional support.

• Added value of Integrated Care for Older People Step1 for identifying pre-frail/frail individuals compared to existing frailty assessment tests

Numerous assessments exist for identifying frail individuals (Buta et al., 2016). Compared to Integrated Care for Older People Step1, these assessments can be complex, require healthcare professionals, and are generally time-consuming, such as the Frailty Index (Rockwood et al., 2005) or the Fried score assessment (Fried et al., 1991). A major advantage of Integrated Care for Older People Step1 is that it can be completed in 10 minutes or less, is freely accessible via a mobile application (Tavassoli et al., 2021), and can be administered by non-healthcare professionals or completed as a self-assessment. This makes it a scalable screening tool for general populations and community-dwelling older adults in primary care settings, in line with WHO recommendations to reach the largest number of people. Among the Integrated Care for Older People domains, mobility and nutrition are most strongly correlated with pre-frailty/frailty, but their low sensitivity limits their utility as standalone screening tools for pre-frailty/frailty as defined by the Fried criteria. Nevertheless, our study demonstrates that it is possible to identify individuals at higher risk of frailty among participants.

• Implications

This study provides simple, practical recommendations for healthcare professionals involved in the Integrated Care for Older People program. Given the high workload and the need to prioritize individuals at greatest risk of functional decline, referral to Integrated Care for Older People Step2 should be primarily offered to participants presenting abnormalities in Step1 related to mobility (chair rise time) and/or nutrition (weight loss and loss of appetite). This approach enables a pragmatic strategy in primary care settings. For future research, collecting participants’ socio-economic status would allow assessment of its role in frailty risk, potentially guiding prioritization of specific populations for early prevention of functional decline. To verify the validity of the Classification and Regression trees modelling, the proposed model should be tested in other populations, such as the INSPIRE-T cohort in Toulouse (Guyonnet et al., 2021). To more accurately identify frail individuals, the study could be repeated by differentiating between pre-frailty and frailty, allowing evaluation of Step1’s utility and potentially examining a gradient of association across three groups: robust, pre-frail, and frail. The ultimate goal of this work is to provide concrete guidance for the practical implementation of the Integrated Care for Older People program during its evaluation (Glasgow et al., 2022)

• Merging Pre-Frail and Frail individuals in the same group

The decision to pool frail and pre-frail participants in this study was based on the concept that pre-frailty precedes frailty and represents a continuum of functional decline (Sezgin et al., 2022). Without adequate interventions, individuals in the pre-frail stage are at risk of progressing to frailty. In clinical practice, interventions targeting pre-frailty are similar to those for frailty (Gené Huguet et al., 2018). At the individual level, impairment in a single Fried criterion (e.g., weight loss) may be as clinically significant as having two or three criteria if the impairment is severe. For many authors, the frailty phenotype is considered a construct of symptoms, and the presence of even one symptom may define the individual as frail. For the purposes of prioritizing Step2 and Step3 of the Integrated Care for Older People program in primary care, distinguishing between pre-frailty and frailty at Step1 is not essential; both should be considered and managed similarly.

• Strengths and limitations

The main limitation of this study is the collection of real-life clinical data by healthcare professionals, proxies, or through self-assessment using diverse methods. This approach may introduce heterogeneity in data collection and result in substantial amounts of missing data, as shown in the flow chart. However, since the objective of this study is to provide guidance for clinical practice, it is advantageous that the data reflect real-world clinical settings. In the results, the sensitivity of the different associations in the Classification and Regression Trees model is consistently 100 %, indicating limited test specificity. Although external validation remains necessary, this study highlights the association between Step1 and frailty, identifying a population at high risk of disability. The low specificity of Integrated Care for Older People domains for frailty limits their utility as diagnostic tests for frailty. Nevertheless, screening for pre-frailty/frailty using ICOPE Step1 warrants further investigation to confirm the consistency of these findings. The strength of this study lies in the large number of participants, allowing for meaningful analyses based on extensive experience with the implementation of the Integrated Care for Older People program in France.

## Conclusions

This study highlights the association between impairments identified in Integrated Care for Older People Step1 and the presence of pre-frailty/frailty in older adults. Participants with mobility and/or nutrition impairments at Step1 should be prioritized for prompt Step2 and Step3 assessments due to their higher risk of disability onset. Thus, Step1 allows healthcare professionals to rapidly identify individuals most at risk of progressing to dependency and to propose the next steps in their care pathway. Given available resources, it is important to continue this line of research while identifying more precise clinical indicators to facilitate implementation of the program in primary care. This approach addresses participants’ needs by providing targeted interventions for the appropriate clinical profile and supports the broader deployment of Integrated Care for Older People program in the community.

## Funding

This work was performed in the context of the IHU HealthAge, which received funding from the Agence Nationale de la Recherche as part of the France 2030 program (reference number: ANR-23-IAHU-0011).

The ICOPE-Care program was supported by grants from the Occitania Regional Health Agency (Region Occitanie and Pyrénées-Méditerranée; reference number 1901175), ICOPE National Experimentation- Article 51 (Ministry of Solidarity and Health - Order of July 19, 2022 - NOR: SPRS2221913A), the European Regional Development Fund (project number MP0022856) and The Interreg Program V-A Spain-France-Andorra (European Union) in the context of the APTITUDE (EFA232/16) and the APTITUDE-PROXI (EFA018/01) projects.

**The funders had no role in study design, data collection and analysis, decision to publish or preparation of the manuscript.**

## CRediT authorship contribution statement

**Caroline Berbon:** Writing – review & editing, Writing – original draft, Methodology, Formal analysis, Data curation, Conceptualization. **Gabor Abellan Van Kan:** Writing – review & editing, Writing – original draft, Validation, Supervision, Methodology, Formal analysis, Conceptualization. **Yves Rolland:** Writing – review & editing, Supervision, Methodology. **Laurent Balardy:** Writing – review & editing, Supervision, Project administration. **Catherine TAKEDA:** Writing – review & editing, Investigation, Data curation. **Néda Tavassoli:** Funding acquisition, Data curation, Conceptualization. **Véronique Bezombes:** Project administration, Data curation. **Bruno Vellas:** Supervision, Funding acquisition, Data curation. **Sandrine Andrieu:** Writing – review & editing, Methodology, Conceptualization. **Maria Eugenia Soto:** Writing – review & editing, Writing – original draft, Supervision, Investigation, Funding acquisition, Data curation, Conceptualization.

## Declaration of competing interest

The authors declare that they have no known competing financial interests or personal relationships that could have appeared to influence the work reported in this paper.

## Appendix Supplementary materials

Image, application 1

Image, application 1
