# Supplementary figures and images for: Identification of pre-frail/frail older adults using the Integrated Care for Older People WHO Step1 screening tool: a cross sectional study
Source: Int J Nurs Stud Adv. 2025 Dec 4;10:100463. doi: 10.1016/j.ijnsa.2025.100463 (PMC12757617; doi:10.1016/j.ijnsa.2025.100463)

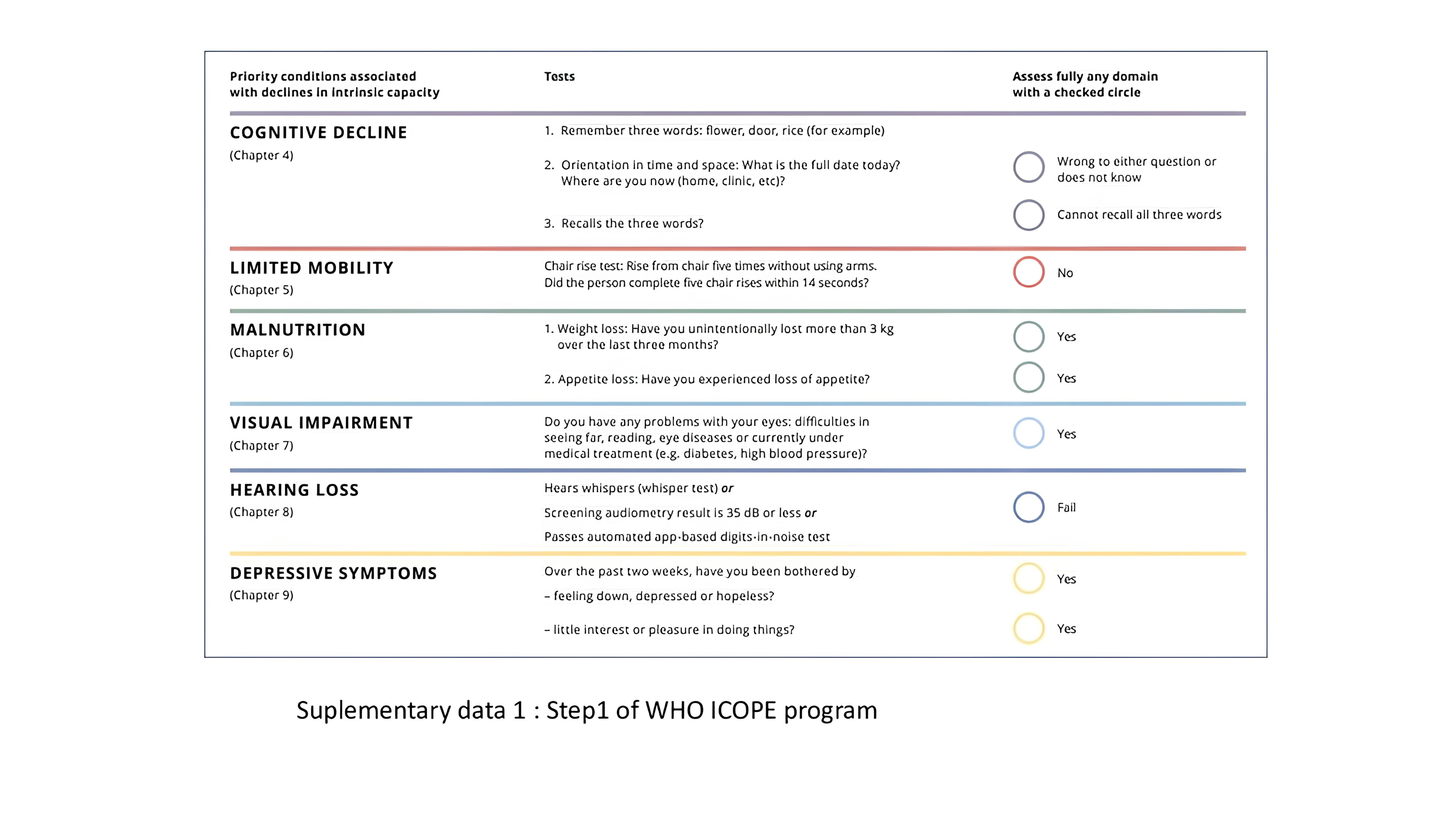

Supplement: Supplementary file 1 [file mmc1.zip › mmc1.jpg]
