# Supplementary material for: Identification of pre-frail/frail older adults using the Integrated Care for Older People WHO Step1 screening tool: a cross sectional study
Source: Int J Nurs Stud Adv. 2025 Dec 4;10:100463. doi: 10.1016/j.ijnsa.2025.100463 (PMC12757617; doi:10.1016/j.ijnsa.2025.100463)
Supplement: Supplementary file 2 [file mmc2.pdf]

C.R.T Muzillac / Elven / Questembert
CH Dordogne
CHU Angers
CHU LIMOGES
CIAS Arlysère
CIR-PA
CNETH
CPTS 13 et 14
CPTS Activ'Santé (Marseille 4-5)
CPTS Adour Chalosse Tursan
CPTS Adour Gave
CPTS AL'AUBE SANTÉ
CPTS Arlysere
CPTS AUNIS NORD
CPTS AUNIS SUD
CPTS Bassin Dacquois
CPTS BOOST UP
CPTS Boucle de Seine
CPTS Centre Vendée
CPTS Cerebellum
CPTS Cœur des Landes
CPTS Colmar
CPTS COMPIEGNE
CPTS Confluence
CPTS COTE D'ARGENT
CPTS Côte Sud Landes
CPTS Coteaux Rhodaniens
CPTS Croix-Rousse Presqu'île
CPTS D'AURAY
CPTS de l'Hautil
CPTS de la métropole nancéienne
CPTS de la Vallée de la Meurthe
CPTS de la Vallée de la Sarre
CPTS de l'Adour
CPTS de Lérins
CPTS de Strasbourg
CPTS de Thiers
CPTS des 2 Baies
CPTS des Baous
CPTS des collines de Valbonne
CPTS des grands Lacs
CPTS du Brivadois
CPTS du Canton Vert
CPTS du Marsan
CPTS du Massif Vosgien
CPTS du Mont-Blanc
CPTS du Pays Bigouden
CPTS du Pays de Quimperlé
CPTS du Pays Salonais

CPTS du Tournugeois
CPTS entre Ceze et Gardon Filieris
CPTS Grand Saumurois
CPTS Haute Lande Armagnac
CPTS Haute-Corrèze
CPTS Ile de Ré
CPTS Itineraire Santé
CPTS La Caravelle
CPTS La Cévénole
CPTS La Rochelle
CPTS Littoral Vendéen
CPTS Lyon 7
CPTS Lyon 8
CPTS Lyon Nord
CPTS Monts du Lyonnais
CPTS Moselle Sud
CPTS Mulhouse
CPTS Nice Nord Collines
CPTS Nice Ouest
CPTS Occitane
CPTS Opale Sud
CPTS Paris 13
CPTS Paris 14
CPTS PAYS CHATELLERAUDAIS
CPTS Pays de l'Etoile
CPTS Pays de Retz
CPTS Pays des Sources
CPTS Pays Fouesnantais
CPTS Porte de France Moselle
CPTS Porte Dromardèche
CPTS Provence Santé
CPTS Sel et Eau
CPTS Sèvre et Loire
CPTS SOLIDAR
CPTS SUD ANGOUMOIS
CPTS SUD COTE D'OR
CPTS Sud Loire Vignoble
CPTS Terre de Chalosse Adour
CPTS Territoire de Guérande / Pays Blanc
CPTS Val d'Artière
CPTS Val de Saone
CPTS Vallée de l’Ubaye
CPTS Vallons du Lyonnais
CPTS Venissieux
CPTS VERTASANTE
CPTS Vignes & Calanques
CPTSde l'Yzeron
CRT AIDOMI Bordeaux
CRT Jean Lachenaud Frejus
CRT LORIENT

DAC 16
DAC 17
DAC 33
DAC 40
DAC 46
DAC 47
DAC 55
DAC 64
DAC 78 Sud
DAC 79
DAC 86
DAC 87
DAC 92 Nord
DAC 93 Sud
EMEIS
Filière gériatrique du Val d'Oust à Brocéliande
FILIERIS Bassin ALES
FILIERIS Carmaux
Gérondif
Gérontopole Normandie
Gérontopole Pays de La Loire
GHT Normandie centre
GHT Val de Seine et Plateaux de l'Eure
Hopital américain
ICOPE 29/ CHU BREST
InterCPTS du LIMOUSIN
IPEP fragilité Centre Manche
Les Lys d'Argent
MAIA Guyane
MSP Ballancourt Santé
MSP de Gerbeviller
MSP Santé&Co' / Le Havre Nord-Est
MSPU Saint Priest
Mutualité Francaise Dignes Les Bains
OSE Paris 12
Paris 12
SSR Les Chatelets
